# Optimization of Vehicular Networks in Smart Cities: From Agile Optimization to Learnheuristics and Simheuristics

**DOI:** 10.3390/s23010499

**Published:** 2023-01-02

**Authors:** Mohammad Peyman, Tristan Fluechter, Javier Panadero, Carles Serrat, Fatos Xhafa, Angel A. Juan

**Affiliations:** 1 Department of Computer Science, Multimedia and Telecommunication, Universitat Oberta de Catalunya, 08018 Barcelona, Spain; 2 Smurfit Business School, University College Dublin, Blackrock, D04 V1W8 Dublin, Ireland; 3 Department of Management, Universitat Politècnica de Catalunya, 08028 Barcelona, Spain; 4 Department of Management, Euncet Business School, 08225 Terrassa, Spain; 5 Department of Mathematics, Universitat Politècnica de Catalunya, 08028 Barcelona, Spain; 6 Department of Computer Science, Universitat Politècnica de Catalunya, 08034 Barcelona, Spain; 7 Department of Applied Statistics and Operations Research, Universitat Politècnica de València, 03801 Alcoy, Spain

**Keywords:** vehicular networks, smart cities, optimization, heuristics, open data

## Abstract

Vehicular ad hoc networks (VANETs) are a fundamental component of intelligent transportation systems in smart cities. With the support of open and real-time data, these networks of inter-connected vehicles constitute an ‘Internet of vehicles’ with the potential to significantly enhance citizens’ mobility and last-mile delivery in urban, peri-urban, and metropolitan areas. However, the proper coordination and logistics of VANETs raise a number of optimization challenges that need to be solved. After reviewing the state of the art on the concepts of VANET optimization and open data in smart cities, this paper discusses some of the most relevant optimization challenges in this area. Since most of the optimization problems are related to the need for real-time solutions or to the consideration of uncertainty and dynamic environments, the paper also discusses how some VANET challenges can be addressed with the use of agile optimization algorithms and the combination of metaheuristics with simulation and machine learning methods. The paper also offers a numerical analysis that measures the impact of using these optimization techniques in some related problems. Our numerical analysis, based on real data from Open Data Barcelona, demonstrates that the constructive heuristic outperforms the random scenario in the CDP combined with vehicular networks, resulting in maximizing the minimum distance between facilities while meeting capacity requirements with the fewest facilities.

## 1. Introduction

The growing global population and preference for urban living have made city management a challenging issue for city planners and policy makers. Modern cities need to adapt to the emerging needs of their citizens [1]. The development of intelligent transportation systems (ITS) is one of the key characteristics of smart cities. ITS aim to improve the efficiency and safety of the road and transportation systems through new applications, protocols, and standards, which allow vehicles to function as a sender, collector, and switch for data broadcasting or multicasting. Furthermore, the growing number of vehicles motivates efforts to improve road safety and inter-vehicle entertainment through vehicular systems [2]. As a result of advancements in wireless technologies and the growing popularity of the Internet of Things (IoT), researchers were able to develop communication systems in which vehicles directly participate in the network. As a result, networks such as VANETs have been proposed to enable communication between vehicles and everything else, as well as between roadside units (RSUs) and people [3].

VANETs can help smart cities by improving vehicle mobility and implementing an efficient system for communicating and managing warning messages. For instance, efficient traffic alerts and up-to-date traffic incident information will reduce traffic congestion, improve road safety, prevent car accidents, and enhance city driving. Additionally, real-time traffic alerting will reduce travel distances, fuel consumption, and, as a result, emissions of CO_2_ [4]. Furthermore, due to the increasing need for communication, computation, and storage resources, emerging vehicular applications, and exponentially growing data, Vehicular Edge Computing (VEC) has great potential to improve traffic safety and travel comfort by bringing communication, computing, and caching resources closer to vehicular users. It could also be able to meet the growing demand for low latency and bandwidth in edge devices [5].

**Figure 1:**
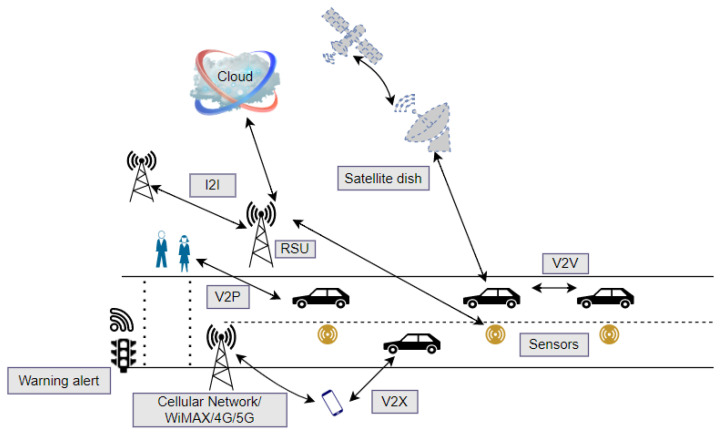
VANET in Smart Cities.

Figure 1 shows VANETs communication in smart cities, where communication can take place between infrastructure-to-infrastructure (I2I), vehicle-to-vehicle (V2V), vehicle-to-infrastructure (V2I), and vehicle-to-everything (V2X) such as people, mobile phones, RFID readers, traffic lights, and so on. The direct communications between devices and vehicles are based on wireless access standards such as 4G, 5G, DSCR, etc. Small sensors installed beneath the asphalt can measure traffic density, generate data, and send it to the open repositories. The RSU is fixed and consists of a transceiver that transmits and receives data. These mobile devices and vehicles are linked to edge devices such as RSUs and share the edge layer. The edge serves as a bridge between the cloud and devices, vehicles, and people. Servers with computational and storage capabilities are deployed close to vehicular networks, and data processing and analysis are performed close to end devices. As computing and storage services are provided close to the user (on the edge), edge computing services provide a better quality of service (QoS).

Since people are consuming more information with their mobile devices, vehicles are equipped with edge devices and RSUs technologies in the road transport network, and the popularity of new mobility services such as ridesharing and carsharing has increased communication between vehicles, people, and everything else [6,7]. Therefore, the information gathered by them can be used to evaluate and predict real-time traffic density and compute an accurate map of road traffic density, as well as assist VANET in improving transportation efficiency, and pedestrian comfort, and provide a QoS. Cloud, fog, and edge computing techniques enable the real-time transmission and processing of terabytes of data. The cloud node has a lot of memory and processing power, but the fog and edge nodes have limited capacity. Additionally, the physical distance between the cloud data center and the fog and edge nodes influences the data transfer rate, and if it is long, it increases latency and potential packet loss. Furthermore, one of the primary goals of VANET is to provide QoS to end users, while infrastructure deployment is the most significant challenge in the traffic improvement application of VANET.

In this context, we combined the Capacitated Dispersion Problem (CDP) and vehicular networks in order to efficiently allocate facilities which can result in proper utilization of all facilities as well as timely reaction, which is required for smart cities, to improve the QoS in VANET. CDP aims at maximizing the dispersion of the open facilities while fixing a given capacity threshold to make facility capacity sufficient to meet customers’ demands. Since CDP is NP-hard, exact methods may take a long time to guarantee the optimality of a solution when dealing with large instances [8]. Furthermore, because approximate methods such as heuristics and metaheuristics have been demonstrated to be effective and capable of producing high-quality solutions for large-scale and complex real-world problems, optimization techniques such as these are now widely used. In particular, heuristics have a strong potential to offer agility and real-time responses, which are critical for an effective ITS [9].

In this paper, we aim at reaching the following goals: (i) to elaborate a comprehensive overview of vehicular networks; (ii) to provide optimization challenges regarding ridesharing, carsharing, VEC, and traffic improvement applications in VANETs; and (iii) to propose a case study, based on real-life data, which combines a CDP and vehicular networks. The organization of this paper is introduced as follows: Section 2 presents an overview of vehicular networks. In Section 3, we provided optimization challenges regarding ridesharing, carsharing, and traffic improvement applications in VANET. We present a case study and computational results in Section 4. Lastly, Section 5 summarizes our main conclusions and provides future research lines.

## 2. Vehicular Networks: An Overview

### 2.1. Vanets: A Conceptual Framework

The notion of networks characterized by a dynamic structure and limited transmission speed and quality is no recent innovation—in their 1999 paper, Corson and Macker [10] coined the term mobile ad hoc network (MANET). These networks are characterized by a set of mobile routers which create routes for information transmission as needed [11]. In ITS, vehicles can use communication technology to counteract and eliminate transportation inefficiencies [12]. VANETs are the extension of this line of thought; vehicles and RSUs act as network nodes that send, transmit, and receive data enabled by a combination of wireless access and network routing technology [13]. These vehicles range from regular roadside transportation to drones [14]. Connectivity in a VANET is naturally quite demanding due to the dynamic behavior of network nodes as vehicles enter, move within, and exit specific regions of the network [15]. One crucial mechanic that VANETs can use to improve network quality is that the path of network nodes is somewhat predictable as vehicles in certain directions on a mobility grid [16]. Ultimately, the interactions between all VANET participants require fast and complete communication to satisfy the ambitions of dynamic mobility systems [17].

Therefore, the arguably most important task to enable VANET-based mobility in smart cities is to ensure a high QoS, which is influenced by the two main forms of communication occurring in a VANET context: First, vehicles transfer information with each other in a peer-to-peer, or V2V manner. Second, vehicles can tap into a flow of data through RSUs either through a direct connection with the RSU or with a relayed connection through a V2V network path [18]. In all communication between nodes in the network, transmission follows one simple rule: Two nodes can only exchange data if they are within broadcasting range of their wireless devices [19]. In most VANET applications, accordingly, nodes greedily forward transmissions by selecting a node in the target direction [20]. Single-hop transmission occurs when the broadcasting node sends information to neighboring nodes; multi-hop transmission requires nodes to re-broadcast information [21]. To ensure an uninterrupted flow of data in multi-hop transmission scenarios, nodes can store information and only forward them once a suitable transfer node is found [22]. Over the last decade, a plethora of routing protocols have been proposed by researchers and compared in regard to their performance [23,24,25,26]. In a 2014 meta-analysis, Dua et al. [18] cluster routing protocols into five predominant groups such as topology-based, geographic, hybrid, clustering, and data fusion. All of these protocols aim to create a network that can withstand the demanding nature of smart city connected mobility. Belamri et al. [27] provide a framework of parameters in regard to which a VANET routing protocol should be optimized: Most importantly, routing quality should be assessed concerning message delay, network node distances, link reliability, hop count, and mobility of nodes. In evaluating the QoS of a network with specific routing protocols, researchers should use network metrics such as end-to-end delay (EED), packet loss, throughput and bandwidth, and packet sending rate (PSR). Following this logic, a VANET routing protocol and its technology should be sufficient to enable whichever application needs to be run in the network. VANET applications for smart city mobility can generally be clustered into one of two applications: efficiency-oriented and safety-oriented optimization [28]. Efficiency-oriented applications are mainly concerned with the overall flow of traffic in a VANET environment. A VANET infrastructure can be used to host a variety of applications such as traffic congestion detection and mitigation [29,30,31], traffic forecasting [32,33], fuel-saving vehicle routing [34,35], or secondary efficiency enabled by internet access, for example, by providing internet during traffic jams [36]. All of these applications prove to be a use case for optimization techniques. Safety-oriented services in a VANET are concerned with vehicular security. Given VANET connectivity, these services can be used to prevent collisions [37,38,39], facilitate emergency service response [40,41,42], or support safe overtaking [43,44].

**Figure 2:**
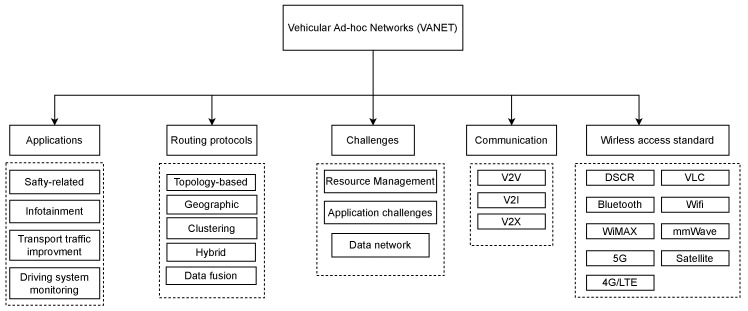
General Operation of VANET.

Figure 2 depicts the general operation of a VANET, which includes applications, routing protocols, challenges, communication, and wireless access standards. Above, we discussed the routing protocol and different communications in VANET. In terms of applications, VANETs can provide a wide range of services and applications. The applicability of these services and applications allows us to classify them into safety-related, infotainment, traffic improvement, and driving system monitoring. Additionally, each of these applications presents challenges for VANET. Other challenges could include resource management, in which resources are shared among vehicles, presenting numerous difficulties for VANET deployment. Since vehicles in the VANET have mobile communication devices and share data, data networking is another challenge in this area. Finally, the expansion of VANET services and the need to ensure the continuity and scalability of VANET communication have motivated the use of various types of wireless communications such as DSCR, 4G, 5G, WiMAX, etc.

### 2.2. Vanets and IoT, Edge Computing

Network routing protocols are one of the two cornerstones of connected smart city mobility. They are enabled by IoT devices that, as the second cornerstone, are the foundation upon which interconnected ITS are built [45]. The IoT paradigm envisions communication between objects that are already part of everyday life to create an infrastructure of devices that are embedded into larger networks [46]. In the context of vehicular mobility, a vast array of use cases have been investigated and implemented in research: IoT devices can be used to reserve and guide vehicles to parking spots [47,48] or to avoid vehicle collisions [49] by providing an exchange of information between network nodes. The challenge of transmitting large amounts of data over a vehicular network has led to research into how external processing and storage could ameliorate the exchange of time-critical information. Data-center facilitated cloud computing can dynamically integrate into a VANET application, allowing network nodes to off-load data-intensive applications [50]. Hussain et al. [51] was the first to propose a cloud-based VANET architecture to connect vehicles and support application loads. These vehicular cloud computing networks allow for scalable network architectures that support the ever-increasing stream of data and help alleviate connectivity limitations [52]. As Shrestha et al. [53] argue, cloud computing might reach its limitations in the context of more demanding VANET environments where large numbers of vehicles demand real-time applications. Consequently, they propose to enhance VANETs with edge computing.

Edge computing further develops the key functionalities of cloud computing by moving data processing units closer to each network node: Calculations to support network nodes are performed at the edge of a network [54]. Garg et al. [55] demonstrate that using edge nodes as an “intermediate interface between network and cloud” in VANETs can indeed improve network latency and facilitate overall data flow. It is important to note that network structures supported by edge computing are not restricted to mobility on the ground; the concept can be extended to any network node in three-dimensional space, such as unmanned aerial vehicles (UAVs) [56,57]. These UAVs can even be used to flexibly support a VANET architecture if needed [14]. Aside from providing a more capable architecture, edge computing also proves to be resistant to network attacks [58]; fast data transfers and processing allow for reliable message verification to ensure no malicious communication occurs in the network [59]. Recent proposals even go as far as integrating blockchain technology to ensure network integrity [60].

**Figure 3:**
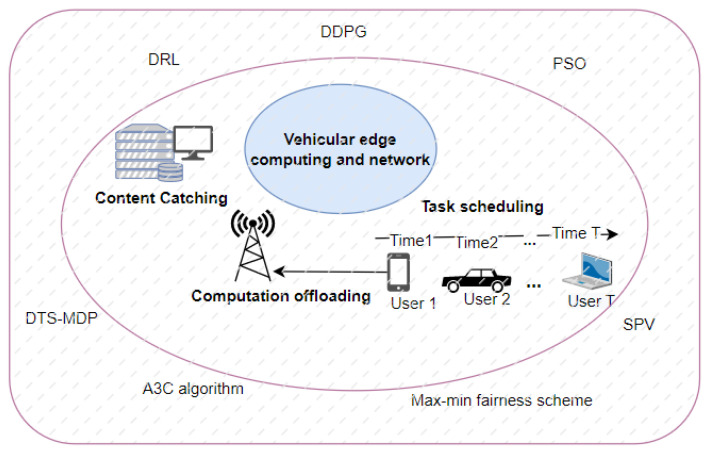
Operation of VEC.

VEC is a promising technology that can be used to support ITS services, smart city applications, and urban computing. Figure 3 shows the problems and methods that are used in the literature reviewed in VEC.

Qi et al. [61] introduced a knowledge-driven (KD) service offloading decision framework for the Internet of Vehicles (IoV), which provides a unique platform for various vehicular services and aims at achieving long-term optimal performance experienced by vehicular users, and based on that, they proposed offloading decisions as a resource scheduling problem with single or multiple objective functions and constraints, where some customized heuristics are used. The framework consists of a decision model which uses deep reinforcement learning (DRL) to learn decision knowledge, and an observation function to obtain vehicular mobility and edge computing node data. To realize online optimization of offloading decisions, they proposed a KD service based on an online A3C algorithm. Evaluating the performance of KD service offloading decisions, they showed that the framework achieves low service delay, can learn the distribution of task data dependency, and almost always chooses a proper destination for large data transmission tasks.

Qiao et al. [62] proposed a new edge caching scheme that optimizes content placement and delivery in VEC and networks with limited storage capacity and bandwidth by taking into account time-varying content popularity, dynamic network topology, and vehicle driving paths. Edge caching was modeled as a double time-scale Markov decision process (DTS-MDP). The joint content placement and the delivery problem is NP-hard long-term mixed integer linear programming (MILP). As a result, the variable participation of vehicles increases the operational complexity of the edge caching system, making it difficult to find the best solution. Thus, they proposed a deep deterministic policy gradient (DDPG) learning algorithm based on a DRL-based cooperative caching scheme to provide low-complexity decision making and adaptive resource management, and they accelerated the learning speed and improved caching performance by using mini-batch gradient descent.

Furthermore, they concentrated on the model-free reinforcement learning approach to provide training guidelines based on a large number of historical experiences. This model-free approach is divided into three categories: critic-model (value-based approach), actor-model (policy-based approach), and actor-critic learning approach, which employs deep neural networks to provide an accurate estimation of deterministic policy function and value function.

As a result, the actor-critic learning framework and the double time-scale content caching model combined to develop a DDPG-based cooperative caching technique. The performance was compared using two benchmark schemes: (i) random caching; and (ii) noncooperative caching. To improve the accuracy of vehicle destination prediction, the destination of the vehicle was predicted using a machine learning model based on shorter strings rather than longer strings to represent the transport region of smart vehicles. The analysis of caching performance based on the DDPG learning algorithm revealed that as the number of episodes increases, all content caching schemes can approach their stable cumulative average cost. The noncooperative caching scheme had the highest average system cost, which includes the cost of content storage and access. In addition, the proposed caching scheme yielded the lowest system cost, and lowest content access latency, and improved the content hit ratio, particularly in the low content delivery latency, when compared to the other benchmark schemes.

Chen et al. [63] proposed a task offloading scheme based solely on V2V communication, based on the gathered period of vehicles in urban environments due to traffic lights or areas of interest (AOI) to minimize task processing time. The Max-Min Fairness scheme is used to optimize the task execution time, which is then solved by the particle swarm optimization (PSO) algorithm. On the one hand, for the special case where all service vehicles participate in task processing, the proposed algorithm provides the optimal solution based on adapted Max-Min Fairness. On the other hand, the PSO algorithm is used for generating a feasible solution for the general case where it is unknown whether each service vehicle will participate in task processing or not. Furthermore, to evaluate the performance of their proposed scheme, they generated vehicle track files using the TIGER map and then used the IDM IM model provided by VanetMobiSim to compare the performance of computed schemes such as local computing, offloading with the Max-Min Fairness Algorithm, and offloading with the PSO Algorithm. The results showed that the Max-Min Fairness algorithm and the PSO algorithm reduced task execution time based on the number of service vehicles used. Additionally, the increasing number of service vehicles showed that the PSO algorithm is slightly better than the Max-Min Fairness algorithm in terms of robustness over different task sizes for each scheme.

Wang et al. [64] considered VEC and networks with dynamic topologies, unstable connections, and unpredictable movements and proposed a near-optimal performance imitation learning-enabled online task scheduling algorithm. In their proposed algorithm, they used the terms service providing vehicles (SPV) and VEC servers interchangeably. Furthermore, the task scheduling problem was considered by minimizing the average consumed energy of offloaded computation tasks while ensuring their execution latency based on SPV clustering and imitation learning approaches.

The branch-and-bound algorithm was used with a few iterations as the expert’s trajectories, and the learning agent made proper task scheduling decisions by mimicking the expert’s demonstrations with the help of imitation learning. They proposed an imitation learning-based task scheduling algorithm that allows the learning agent to make timely scheduling decisions instead of global searching, which is time-consuming and computationally intensive and is not suitable for online scheduling. To validate the performance of their proposed algorithm, they compared it to four other designed algorithms: the Deep Q Network (DQN)-based algorithm, DATE-V, local optimization, and FORT. As a result, the proposed algorithm’s average energy consumption was much lower than that of the other algorithms, and its task-processing ratio was higher than that of the other four algorithms.

## 3. Optimization Problems in Vehicular Networks

### 3.1. Ridesharing

**Figure 4:**
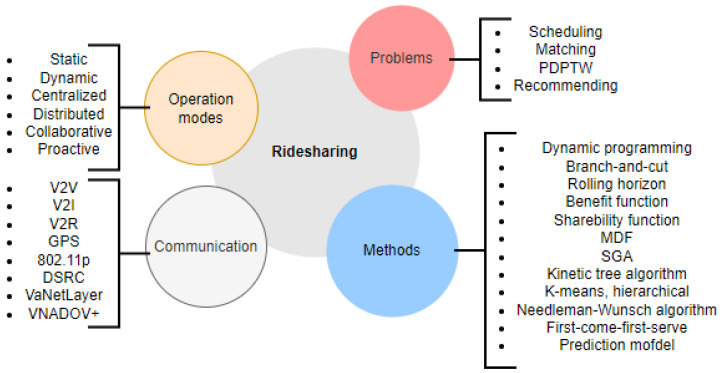
Operation of RideSharing.

Ridesharing in public/private vehicles is an intriguing problem that has piqued the interest of numerous researchers. The taxonomy, shown in Figure 4, divides the literature discussed in this section into four categories: operation modes, problem type, methods used to solve the problem, and type of vehicle communication/protocol used.

There are two types of ridesharing: static and dynamic. Static ridesharing assumes that driver and rider requests are known before executing a matching process, attempting to cover a wide variety of types such as dial-a-ride problems (DARP), carpooling, and slugging. Due to the complexity of ridesharing optimization, researchers use or adopt various heuristics, as well as mixed-integer/integer linear programming models, to solve large-sized instances [7]. Dynamic ridesharing is a service that dynamically arranges ad hoc shared rides, made possible by low-cost geo-locating devices, smartphones, wireless networks, and social networks [65]. The dynamic ridesharing problem focuses on the fact that passenger requests are generated in real time. In this regard, Huang et al. [66] proposed a branch-and-bound algorithm to solve the problem of ridesharing with service guarantees on road networks; moreover, they proposed a kinetic tree algorithm to better schedule dynamic requests and adjust routes on the fly. Their flexible algorithm could also handle changing road network layouts and traffic conditions. It creates a server trip schedule based on the server’s location upon request, calculating trip cost between any two points on the schedule, and satisfying point order, waiting time, and service constraints. Furthermore, they build an augmented valid trip schedule that combines new requests with existing ones to share a partial trip among customers. Using their proposed kinetic tree approach, they allow constraint flexibility if pickup and drop-off locations are close to each other.

Based on dual social group architecture (SGA), Zhao et al. [67] proposed a distributed ridesharing service that divides messages into driver social group architecture (DSGA) messages which include a driver’s destination, and vehicle social group architecture (VSGA) messages that provide information on traffic condition. Assigning a number of token vehicles to each vehicle, tokens in the beacon packet were transmitted to the relay vehicles’ neighbors by the relay vehicles. Neighbor vehicles will first collect the feature-level atomic messages with a one-hop communication scope and then fuse them using a fuzzy cluster method to generate the feature-level result. Each relay vehicle receives this message from the token vehicle and generates its own VSGA messages; this methodology allows to determine traffic conditions in double vehicle communication distance. Through this dual-SGA methodology, passenger wait times are significantly reduced.

Bathla et al. [68] proposed four different ridesharing system models based on the pickup and drop-off locations of potential riders. They also proposed a dynamic algorithm for models in which the pickup and drop-off locations are different for all users. With DBSCAN clustering, they simulated the scenario of multiple ridesharing requests from the same or nearby regions. Grouping together requests and calculating the distance between two locations using the Haversine formula and the Google Maps Direction API, they divide the cost for passengers with shared ride distance evenly. They assessed the algorithm using ridesharing metrics such as satisfying requests and waiting time per passenger. Additionally, they implemented a taxi distance minimization algorithm with a complexity of O(n×m) where *n* and *m* are pickup and drop-off events, finding that their proposed algorithm accommodates higher ridesharing among passengers.

Alisoltani et al. [69] concentrated on the automatic matching process, which is one of the most difficult challenges in dynamic ridesharing. They used a variety of techniques, including exact methods based on branch-and-cut and the rolling horizon method to solve the problem dynamically for quality of solution; an AI-based technique to limit the number of requests for the solver; a clustering method such as K-means and hierarchical clustering based on the shareability function to place the most shareable trips within a separate cluster; and finally, a heuristic algorithm to solve the matching problem within each cluster. As a result, the final algorithm provided high-quality solutions for large-scale problems in a short amount of time. It considers both passengers’ and service providers’ objectives, minimizing total travel time and distance while also minimizing passenger waiting time. To simulate the operation of their proposed dynamic ridesharing system, the authors created a plant model using macroscopic fundamental diagrams (MFD) to simulate real-world traffic conditions and a prediction model which calculates travel times during the assignment process using mean vehicle speed.

Aydin et al. [70] proposed a new ride matching algorithm that takes into account the participants’ characteristics and preferences. They defined joint socialness score (JSS) as a score of similarity between a driver and a rider while maintaining the maximum number of participants in a ridesharing system, and they planned to maximize JSS and allow a driver to be matched with more than one rider, even if only single rider-single driver matches are permitted. They checked the similarities between the routes of the drivers and riders using the Needleman–Wunsch algorithm and specified the score of matching, mismatching, and gap. Then, it was modified by removing the traceback process. They used the first-come, first-served method for matching. As a result, when a rider enters the system, the feasibility of each available driver is checked first. Following that, the JSSs for all possible drivers are computed. The rider is paired with the driver who has the highest corresponding JSS. The computational burden imposed by splitting drivers’ routes on the algorithm resulted in longer computation times. Additionally, the proposed heuristic finds matches on relatively short notice, compared to integer programming, and also can be used to solve more complex and large-scale problems.

Unlike most vehicular applications, which rely on the availability of an easily accessible internet infrastructure, Bravo-Torres et al. [71] focused on advanced services deployed by VANET to vehicles without infrastructure access. Their proposed multi-layer architecture is built on a procedure combining request, response, and acknowledgment messages with timers. A knowledge management layer facilitates the modeling of locally stored user profiles, using context-aware algorithms to match potential riders’ mobility needs based on their itineraries and preferences. The route matching algorithm defines two distinct methods based on Euclidean distances: one that detects regular user routes using past itineraries, and one to determine whether two users can share a route based on weighted user characteristics. Both methods use GIS technologies to locate routes on a map and model user mobility patterns. Testing their proposal in a VANET simulator using the Simulation of Urban Mobility (SUMO) software to model traffic and NS-3 to simulate communications, they found that their VaNetLayer significantly reduced downtime and increased savings, outperforming the AODC and VNAODV protocols for delivery ratios.

Olakanmi and Odeyemi [72] proposed a novel 1-to-n ridesharing scheme for effective ridesharing capable of collaborative 1-to-n ridesharing and recommending the shortest routes and pickup points for riders and drivers based on previously visited locations. Records for this 1-to-n ridesharing scheme are divided into three stages: trust and similarity models, 1-to-n ridesharing shortest routes and pickup points recommendations, and mutual authentication between the rider and car owner. Based on the location records visited, they developed an algorithm that recommends pickup points for riders and the shortest routes for car owners. They examined the efficiency and cost of the proposed scheme in terms of mean waiting time (MWT), capacity overshoot, and computational cost of the proposed mutual authentication. According to the results, the scheme’s mutual authentication procedure had the lowest computational cost when compared to other authentication schemes such as SAMA, ECPP, CAS, GSB, KPSD, and IBCPPA. This reduces the proposed scheme’s service-delay, as evidenced by the insignificant increase in mean waiting time as the number of requests increases.

### 3.2. Carsharing

**Figure 5:**
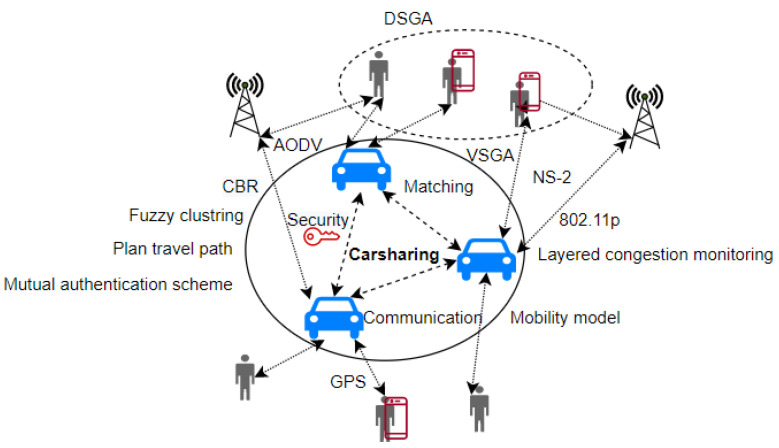
Operation of Car Sharing.

Carsharing offers a flexible alternative that meets a wide range of transportation needs around the world while mitigating the negative effects of private vehicle ownership. Extensive research has been conducted in the field of carsharing systems in recent years, which includes analyzing carsharing trip characteristics, evaluating its impact on society and the environment, and optimizing systems [73]. Figure 5 depicts the literature reviewed in this section on carsharing optimization via vehicular networks.

Zhao et al. [74] proposed a carsharing service in VANET based on a dual SGA in order to improve the quality and robustness of carsharing services while also reducing passenger wait times and avoiding traffic congestion. After a successful match, the vehicle will respond to carpool matching requests via relay vehicles. The DSGA procedure will first calculate the geographic matching based on the driver and passenger destination correlation when the relay vehicle receives the request. The vehicle will then conduct a VSGA identity check. If the match fails, the request is forwarded by the relay vehicle. In the final match step, each relay vehicle merges and collects its traffic data with the help of nearby vehicles via the beacon package. They then distribute a certain amount of tokens to each relay vehicle to control the peripheral congestion message. The authors also proposed a layered congestion monitoring method to collect congestion information and improve matching accuracy. In this process, the relay vehicle first distributes some tokens to its neighbors, and then the neighbor vehicle that received the tokens collects the atomic congestion message in its driving region, such as acceleration, speed, and brake frequency, and then performs fuzzy clustering on the message. The fuzzy clustering method can reduce information and computation redundancy by extracting key information.

Lu et al. [75] combined vehicle mobility simulation and vehicular communication networks to create carsharing systems. They provided two models, one for taxi systems and one for the VANET configuration in NS-2. They used SUMO to generate traffic. Furthermore, classic microscopic traffic theory was used to develop the car following model, which calculates each car’s trajectory to analyze its performance. They chose the ad hoc on-demand distance vector (AODV) routing protocol for the network layer after comparing AODV, DSR, and DSDV. They examined three parameters in carsharing application performance using a Manhattan map generated by TIGER: PSR, Maximum connectivity number (MCN), and vehicle count. As a result, the carsharing performance focuses on two scenarios: the impact of different PSR and MCN on communication performance when the number of cars remains constant and the impact of different numbers of cars when network parameters remain constant. They conclude that improving VANET performance in carsharing systems is possible with a greater number of equipped vehicles and proper control over the maximum connectivity number.

Olufemi and Adedamola [76] improved user service delivery by proposing an effective anonymous authentication scheme capable of detecting and preventing malicious entities from disrupting the carsharing system’s operations. The scheme also includes a conditional identity-tracing approach for tracking and exposing a malicious entity by revoking the misbehaving entity’s privacy. Their proposed scheme includes five entities: an autonomous vehicle taxi, a taxi user, an autonomous vehicle taxi service provider, a trusted registration center, and a taxi call roadside unit. Each entity in the carsharing system registers with a trusted registration center, which generates an entity-specific pre-private key. The entity later upgrades the pre-private key to a private key. To request a service, both the requester and the requestee must perform mutual authentication, which is based on a two-way parameter exchange technique and consists of five phases: setup, registration, mutual authentication, service request, and conditional privacy tracing. In terms of security analysis, they introduced eight theorems with proofs based on a bilinear map. Non-key and key-based hash functions are used to obtain fundamental security against impersonation, collusion attacks, privilege escalation, man-in-the-middle, forward secrecy, and insider attacks. They evaluated the scheme’s computation latency by simulating the computational overhead of the cryptographic operations used to determine the proposed scheme’s verification delay. When the scheme’s verification delay was compared to the verification delays of existing certificate and signature-based authentication schemes, it was discovered that their scheme had the lowest computational cost.

### 3.3. Optimization Challenges in Traffic Improvement Application of Vehicular Networks

**Figure 6:**
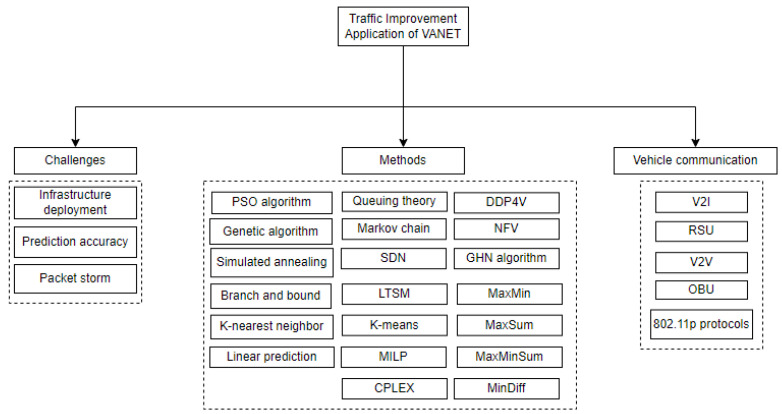
Operation of traffic improvement application.

VANETs promise to improve transportation efficiency, accident prevention, and pedestrian comfort by providing a variety of services and applications to drivers and travelers. These services and applications can be classified as safety-related, infotainment, traffic improvement, or driving system monitoring applications based on their applicability [77]. Figure 6, shows the taxonomy of the literature reviewed in this section on the traffic improvement application of VANET.

There are several challenges to overcome in order to optimize road traffic and reduce travel times by avoiding traffic congestion. One of the difficulties concern infrastructure deployment, where UAVs can act as flying RSUs, relaying data to vehicles outside the RSUs’ coverage range. In this context, a collaborative network coverage enhancement scheme was proposed by Islam et al. [78] to bring these uncovered vehicles within the infrastructure’s coverage. The PSO algorithm was used to determine the best positions to deploy the UAVs, taking into account factors such as vehicle density, heading direction, and previous coverage information. The new positions of the dispatched UAVs were calculated after each time frame, and the UAVs were instructed to move to these positions. Using the traffic simulation tool SUMO, the authors compared the performance of their proposed scheme to other UAV-assisted VANETs schemes, including those without UAVs, fixed UAV-assisted VANETs, and hovering UAV-assisted VANETs, in terms of PDR, hop counts (HOPs), EED, and throughput. The results showed that their proposed scheme outperformed its competitors in the simulation of Daegu, South Korea.

Deploying RSUs in urban areas can be a complex task due to the high cost of installing them at intersections and the large number of possible combinations when there are many intersections. To address this issue, Lehsaini et al. [79] used various metaheuristics, including genetic algorithms (GA), simulated annealing (SA), and improved versions of these algorithms, to determine the best approach for achieving high coverage rates on roads in the target area while deploying a minimum number of RSUs at intersections. The GA-Basic approach includes a probability of performing a mutation operation, where two bits are chosen randomly, while the GA-Improved approach focuses on individuals that increase the overlap of coverage areas. The SA-Basic approach generates an initial solution randomly, while the SA-Improved approach generates it after a preprocessing step that avoids placing RSUs at closely spaced intersections. The authors used the OMNeT-5.0 and SUMO simulators to evaluate the routing performance in terms of PDR and EED based on the number of RSUs deployed in the urban area. The results showed that the GA-Improved approach required fewer RSUs and provided better routing performance in terms of PDR and EED compared to the other approaches.

Additionally, various recent studies address different diversity problems, where Parreno et al. [80] presented mathematical formulations for combinatorial optimization such as MaxMin, MaxSum, MaxMinSum, and MinDiff and solved the problem using the commercial CPLEX solver. Martí et al. [81] proposed new instances, tested them through computational experiments, and demonstrated how these problems have evolved over time from an operations research standpoint. Moreover, based on other previous works Martí et al. [82] formulated a new MILP model to propose an exact and heuristic approach to solve the CDP and later Gomez et al. [83] proposed a BR algorithm that uses the construction-destruction concept to generate high-quality solutions for the CDP in short computing times.

Furthermore, Cao et al. [84] proposed an RSU optimized deployment scheme as a multi-objective optimization problem for mathematical modeling based on large vehicle data, which improves the quality of time-sensitive services while also reducing deployment costs. They proposed a two-step solution in which they obtained the initial RSU deployment location based on road topology and analyzed big vehicle data. They also used the K-nearest neighbor algorithm to remove the overlapping intersection of bidirectional lanes based on the actual road topology situation. Later, the branch-and-bound algorithm was used to achieve optimal RSU deployment. According to the results, the proposed scheme used a small number of RSUs to achieve high coverage.

Another challenge is prediction accuracy, where knowing about potential traffic problems can aid in congestion relief and road capacity expansion. Based on collected vehicular data and the Continuous Time Markov Chain (CTMC), El Joubari et al. [85] developed traffic behavior in multi-lane roads and near intersections. In order to analyze system performance, the queuing theory was used to describe urban traffic dynamics, and CTMC in continuous time was used to forecast long-run average quantities such as congestion rates and average waiting times. Long-term estimates of traffic distribution can be obtained using this method, which employs a numerical method for solving the stationary distribution. In order to validate their model, the results were compared to a queue-based model and realistic traces. The numerical results show that the model accurately reflects real-world urban traffic behavior when historical traffic data are used.

Bhatia et al. [86] presented a VANET system with software-defined networking (SDN) for forecasting traffic flow behavior using computationally intelligent models. They proposed an architecture made up of RSUs and OBUs that is managed by an SDN controller framework and is linked to cloud infrastructure for real-time data storage and high computational capacity. They used a three-phase algorithm, including a configuration phase, a clustering phase, and a running phase, to identify the system’s congestion-sensitive spots before implementing a machine learning model to learn traffic patterns for each spot. They also used the K-means algorithm to find three-dimensional spatiotemporal clusters, which were then processed to finalize the congestion-sensitive spots under a specific RSU. They used the LSTM recurrent neural network architecture to learn time series with long-term traffic flow dependency on the identified congestion-sensitive spot. In addition, detailed and precise LSTM hyperparameter tuning was performed to finalize the set of optimal hyperparameters required for convergence to an optimal traffic flow prediction solution. The results showed that their proposed method can predict future densities with an accuracy of 97% on the entire dataset.

Another issue to consider in traffic management applications are packet storm problems where VANET sends warning messages to vehicles near congested roads in order to keep drivers informed of road conditions and provide the best possible routes to their destinations. This generates a significant number of alert messages, which may cause network congestion and QoS breakdown. In this context, Rizwan et al. [87] proposed a simulation model to reduce broadcast storms by reducing redundancy. Based on three factors—position, distance, and orientation, they developed the next forwarder vehicle (NFV) protocol and, by using the DDP4V technique, analyzed each of these features. The proposed protocol reduced broadcast storms by using DDP4V NFV isolation known as wagon wheels to select the next forwarding vehicle, which can transport data packets 60% faster. In addition, when compared to AID and DBRS, DDP4V has fewer dispersed packets, which reduces retransmissions, and it outperforms standard techniques in high-traffic areas. Considering message transmission results in unwanted data flooding, which causes broadcast storm issues, affecting the overall reliability and performance of VANET. To efficiently minimize the broadcast storming problem, Velmurugan and Leo Manickam [88] proposed a relative speed-based dynamic broadcasting GHN algorithm for broadcasting safety and warning messages in the VANET. For data transmission, the GHN algorithm employs the selective distance allocation methodology. They compared it with SODAD and ABIN, and the results demonstrated that the GHN algorithm can reduce the broadcast storm by more than 2% when compared to the existing algorithm. The system’s output proved to be more efficient in terms of data, throughput, and packet delivery ratio.

Table 1 summarizes the various approaches of the reviewed literature, as well as the objective and specific methods used for various types of problems. Each issue made use of various vehicle communications and protocols, which have also been mentioned.

## 4. A Case Study

We present a case study to show how the CDP described by Gomez et al. [83] can be combined with vehicular networks in real-world scenarios to efficiently allocate facilities in a city. This study aims to maximize the minimum distances between any pair of open facilities where each facility has a known capacity and the total capacity of open facilities must exceed a user-defined threshold.

The CDP can be defined more formally on a complete, weighted, and undirected graph G(V,E), where *V* is a set of facilities and *E* is the set of edges connecting these facilities. Each edge (i,j)∈E has a distance $d_{i,j}>0$ that satisfies the triangle inequality, considering i,j∈V, with i≠j. All distances are symmetric, i.e., $d_{ij}=d_{ji}$. Each facility i∈V has a predetermined, known capacity $c_{i}>0$. As a threshold, collected service capacity *b* is needed. The CDP’s goal is to find a subset O⊂V of facilities with a collected capacity greater than *b* and maximized the shortest possible distance between any two facilities i,j∈S. The threshold is a minimum collected capacity *b* that represents a portion *m* of the total facility capacities $b=m\cdot \sum_{i\in V}c_{i}$.

**Figure 7:**
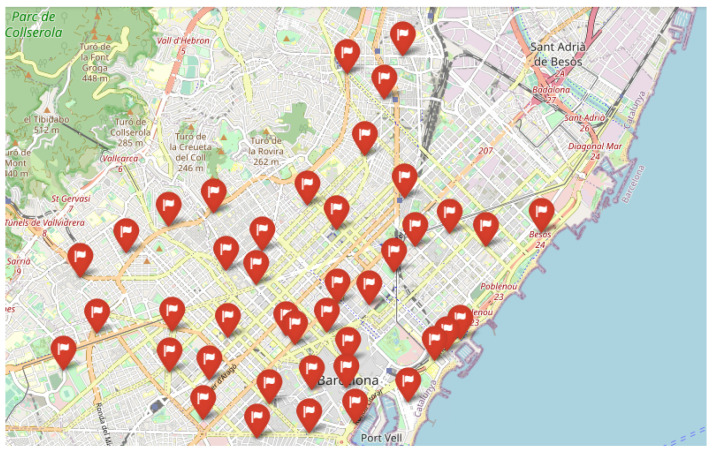
Potential Facilities Location.

The data used to test are obtained from Open Data BCN, specifically, bicing station in the city of Barcelona (https://opendata-ajuntament.barcelona.cat/data/en/dataset/bicing/resource/f59e276c-1a1e-4fa5-8c89-8a8a56e56b34, accessed on 26 December 2022) is used to model CDP. The dataset contains 496 unique locations of bicycle stations and 45 of them, particularly for the electric bicycle charging station. The distribution of electric bicycle charging stations throughout the Barcelona metropolitan area determines the facility’s locations, and the capacity of each facility is the capacity of each electric bicycle charging station. Figure 7 depicts the distribution of potential facilities concerning red flags, and each facility has a known capacity. According to the traffic data (https://opendata-ajuntament.barcelona.cat/data/en/dataset/trams, accessed on 26 December 2022), the traffic situation is classified into various states, such as no data, very-fluid, fluid, dense, very-dense, congestion, and cut-off. Here we consider very-fluid, fluid, dense, and very-dense situations and apply different sizes of threshold *b* to evaluate the performance of Gomez et al. [83] constructive heuristic model in a realistic scenario where the solution is constructed by adding promising elements one by one until the required capacity was reached and compare it to a random scenario where the solution is randomly selected from a list of elements until all the required capacity is covered.

**Figure 8:**
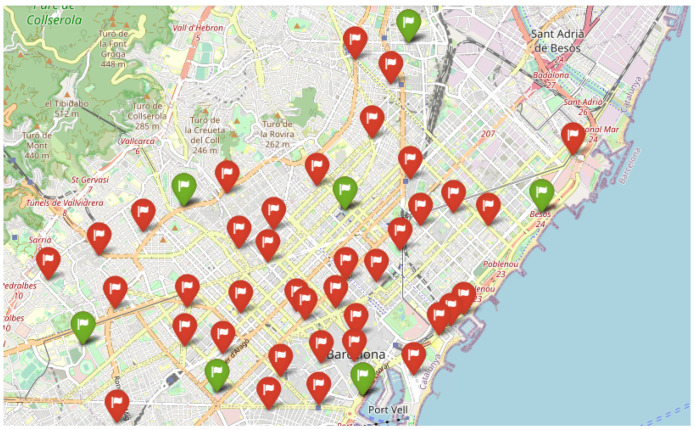
Very Fluid, 7 Selected Facilities.

**Figure 9:**
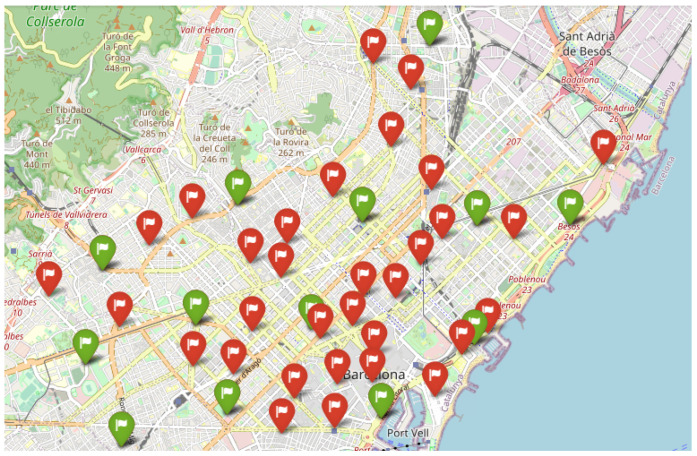
Fluid, 13 Selected Facilities.

**Figure 10:**
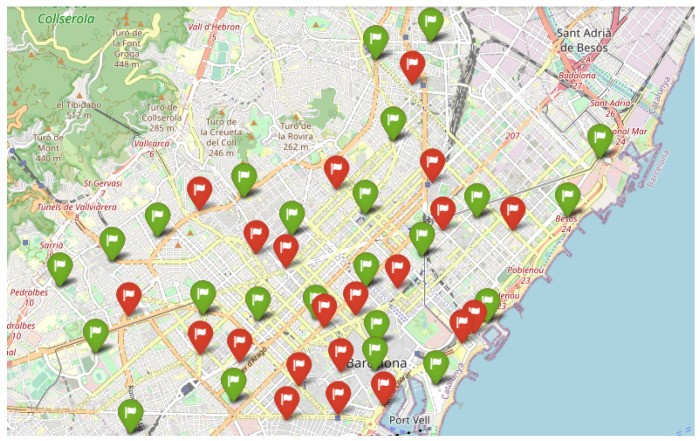
Dense, 24 Selected Facilities.

**Figure 11:**
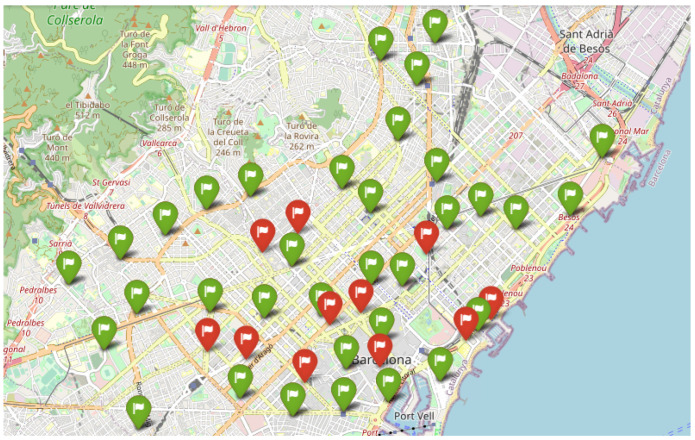
Very Dense, 34 Selected Facilities.

In this section, we demonstrate the outcome of a real numerical experiment using the constructive heuristic methodology Gomez et al. [83], which we then compared to a random scenario. The experiment was conducted on a standard computer. A single instance with 45 unique facility locations and a different capacity threshold was run 30 times with different random seeds. Each threshold is defined on *m* percentage of total capacity and m∈{0.2,0.4,0.6,0.8}, based on the city’s traffic state, where 0.2 for very fluid, 0.4 in a fluid state, and 0.6, 0.8 for the dense and very dense situation. Figure 8 depicts the outcome of employing the constructive heuristic in a highly dynamic traffic situation. In this situation, where there are fewer devices to connect to the facilities, the constructive heuristic algorithm used seven facilities that are not particularly close to one another to cover the required capacity. However, as the situation becomes fluid, the number of facilities chosen increases Figure 9. Figure 10 and Figure 11 show that the algorithm chose 24 and 34 facilities in the dense and very dense states, respectively, to cover the entire required demand while maximizing the minimum distance between each of them.

Furthermore, in order to evaluate the effectiveness of the constructive heuristics in real scenarios, Table 2 compares the performance of the random scenario and the constructive heuristic scenario with various thresholds based on the city’s traffic situation. The instance was run 30 times with different random seeds and the computational time of both algorithms was less than one second. The gap is calculated as Gap=(random_scenario−constructive_scenario)/random_scenario, since the goal of CDP is to maximize the minimum distance between each pair of facilities while meeting the required capacity, a negative distance gap indicates that the algorithm performed better. Table 2 shows a generally better performance of a constructive scenario. Since both scenarios had almost the same demand to cover, the constructive procedure used less facilities to cover all required capacities and maximized the distances between each pair of facilities when compared to the random scenario. The greatest differences in the average distance are achieved in a very fluid (−43.03%) and fluid (−26.22%) state where the constructive scenario utilized 7 and 13 facilities to satisfy the required demand with the average distances of 3127.12 and 1521.98 m, respectively, while the random scenario used 10 and 19 facilities with the distance of 2186.30 and 1205.78 m. Additionally, the gap decreased to −19.12% and −4.24% when the traffic state shifted to the dense and very dense situation. When the traffic state changes from very fluid to very dense, a general decreasing trend in solution quality is identified, requiring a high capacity percentage to open more facilities. As a result, the constructive heuristic outperforms the random scenario in terms of meeting the required capacity with the fewest facilities while maximizing distances between them.

**Figure 12:**
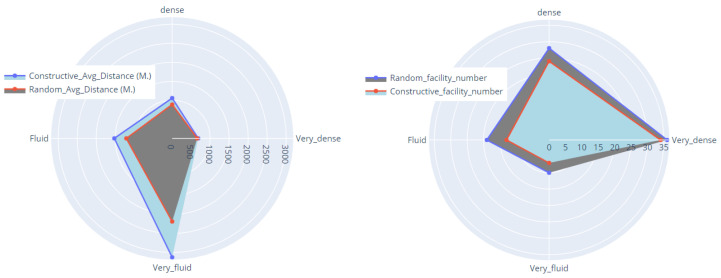
Performance of different scenarios.

Figure 12 depicts the differences between the random and constructive scenarios, demonstrating that the constructive version clearly outperforms the random version in terms of distance and selecting the number of facilities.

## 5. Conclusions

In this paper, we discussed the concept of VANET and the existing challenges in this area, we provided a detailed taxonomy for ridesharing, carsharing, VEC, and the traffic improvement application in vehicular networks, where various challenges were mentioned and a detailed solution was discussed. Furthermore, in order to demonstrate the effectiveness of using agile optimization algorithms in the concept of VANET. We combined state-of-the-art algorithms with vehicular networks, using real data from Barcelona’s open data repository to solve the CDP using constructive heuristics because the constructive approach is built by adding promising elements one by one until the required capacity is reached. Different values are considered for the threshold capacity based on the city’s traffic level, which was determined as a proportion of the network’s total potential capacity. In order to demonstrate the efficacy of the constructed heuristic in a real-world scenario, we evaluated its performance and compared it to a random scenario. As a result, the constructive heuristic outperforms the random scenario by maximizing distances between facilities while satisfying the required capacity with the fewest facilities. Additionally, increasing the traffic volume from very fluid to very dense resulted in a general downward trend in solution quality and necessitated a high capacity percentage in order to open more facilities. Consequently, using constructed heuristic would improve QoS in VANET by making better use of available resources.

In the future, a comparison of this work to the state of the art will be considered. We can also extend it by using predictive models, i.e., machine learning models, to predict facility capacity rather than predetermined capacity and by providing a dynamic model where the threshold changes based on the dynamic situation of the environment. Furthermore, we would like to use a simulator to simulate a realistic scenario of transportation systems with V2X communication and an intelligent roadside unit in order to evaluate the model’s ability to respond adequately to edge node mobility.

## Figures and Tables

**Table 1:** Summarized reviewed work.

Article	Year	Objective	Methods	Type	Vehicle Communication	Case Study
[61]	2019	Obtain optimal offloading policy	A3C algorithm, DRL	offloading decision VEC	WLAN	-
[62]	2019	Minimize storage cost on large time scale	Joint optimization problem DDPG, DTS-MDP, (DRL)	Content caching VEC	V2V, RSU	-
[63]	2020	Minimize task processing time	Max-Min Fairness scheme, Particle Swarm Optimization (PSO)	Computation offloading VEC	V2V, 802.11p, WBSS	-
[64]	2020	Minimizing system energy consumption satisfying task latency constraints	SPV clustering, imitation learning approaches, branch-and-bound algorithm	Online task scheduling	RSU	Hangzhou, China
[66]	2013	Schedule requests in real time, minimize the servers’ traveling times	Kinetic tree algorithm the slack time algorithm, hot-spot clustering algorithm	Dynamic ridesharing schedule problem	-	Shanghai, China
[67]	2014	Minimizing passenger waiting time	Social group architecture, Geometry Matching Method, fuzzy clustering	Distributed ridesharing matching problem	V2V, 802.11p, GPS	-
[68]	2018	Minimize total distance traveled maximize occupancy	Pickup and drop off in different state. Taxi Distance Minimization Algorithm, DBSCAN clustering	Distributed DTRS scheduling problem	GPS	Shanghai, China
[69]	2022	Minimizing waiting time and travel distance clustering shareable trips	Exact method based on branch-and-cut Rolling horizon for dynamic situation k-means, hierarchical clustering plant model and prediction model	Real-time ridesharing matching problem	GPS	Lyon city, France
[70]	2020	Optimizing matches by considering characteristics Maximizing a joint socialness score	Needleman-Wunsch algorithm, Matching process with first-come-first-serve	Ridesharing matching problem	-	Istanbul city, Turkey
[71]	2016	Serve the users’ mobility needs without their active participation	Application layer, knowledge management layer, distribution layer, and an ad-hoc communications layer	Proactive ridesharing	VNADOV+, 802.11p PHY/MAC	-
[72]	2021	Match a minimum number of riders with drivers	Trust and similarity models, 1-to-n ridesharing shortest routes and Pickup points recommendation	Recommending the shortest routes and pickup points for ridesharing	VaNetLayer	-
[74]	2014	Minimizing waiting time	VSGA, DSGA, Fuzzy fusion, fuzzy clustering	Carsharing matching problem	SGA, 802.11p	-
[75]	2013	Evaluate inter-communication system in carsharing	SUMO classic microscopic traffic theory NS-2, CBR	Carsharing communication	AODV, 802.11p	Manhattan map
[76]	2020	Improve service delivery in carsharing minimizing waiting time	Mutual authentication scheme for multi-provider-based carsharing two-way parameter exchange technique	Car sharing security and privacy	Cryptography operations	-
[78]	2022	Find the optimal place for UAVs to minimize the wastage of UAVs service time	PSO algorithm OpenStreetMap, SUMO	Infrastructure development in VANET	V2I, RSU, AODV	Daegu, South Korea
[79]	2022	Minimize the number of RSUs deployed find the best locations	Genetic algorithms Simulated annealing OMNeT-5.0, SUMO	Infrastructure development in VANET	V2V, V2I, 802.11p	Abou Tachfine, Tlemcen
[80]	2021	MaxMin, MaxSum, MaxMinSum, MinDiff problem	Mathematical formulation Comercial CPLEX	Diversity problem	-	-
[81]	2021	MaxMin, MaxSum, MaxMinSum, MinDiff problem	Operation research new instances computational experiments	Diversity problem	-	-
[82]	2021	Maximizes the dispersion of the open facilities	MILP heuristic	CDP	-	-
[83]	2022	Maximizes the dispersion of the open facilities considering the capacity	Biased-randomized algorithm construction-destruction heuristic	CDP	-	-
[84]	2018	Improve quality of time sensitivity services minimize deployment cost	Branch and bound K-nearest neighbor OSMNx, OpenStreetMap	Infrastructure development in VANET	RSU, V2I	Beijing, China
[85]	2022	Stochastic mobility model for urban environments	Queuing theory Markov chain	Prediction accuracy in VANET	RSU, V2I, V2V	-
[86]	2020	Forecasting traffic flow behavior	SDN, LTSM, K-means	Prediction accuracy in VANET	RSU, OBU, V2I, V2V	-
[89]	2019	Minimize total driving and waiting time	Linear prediction OMNeT++, SUMO	Prediction accuracy in VANET	RSU, V2I, V2V, 802.11p	-
[87]	2022	Minimize broadcast storms problem	DDP4V, NFV	Packet storm problem in VANET	V2I, V2V	-
[88]	2019	Minimize broadcast storms problem	GHN algorithm	Packet storm problem in VANET	-	-

**Table 2:** Comparative results between constructive scenario and random scenario.

**State**	**Random Scenario**		
	**Avg_Distance (1)**	**Avg_Capacity (2)**	**Avg_Facility_Number (3)**
Very_fluid	2186.30	56	10
Fluid	1205.78	111	19
Dense	889.56	164	28
Very_dense	658.34	220	36
	**Constructive Scenario**		
	**Avg_Distance (4)**	**Avg_Capacity (5)**	**Avg_facility_Number(6)**
Very_fluid	3127.12	55	7
Fluid	1521.98	108	13
Dense	1059.61	163	24
Very_dense	686.28	216	34
	**Gap**		
	**Gap (1)–(4)**	**Gap (2)–(5)**	**Gap (3)–(6)**
Very_fluid	−43.03%	1.79%	30.00%
Fluid	−26.22%	2.70%	31.58%
Dense	−19.12%	0.61%	14.29%
Very_dense	−4.24%	1.82%	5.56%

## Data Availability

The data used in this study are obtained from Open Data Barcelona.

## Abbreviations

The following abbreviations are used in this manuscript:

AO	Agile optimization
AODV	Ad-hoc on-demand distance vector
A3C	Asynchronous advantage actor critic
AOI	Areas of interest
BR	Biased randomization
BSS	Between-cluster sum of squares
CDP	Capacitated dispersion problem
CAS	Certificateless aggregate signatures
CBR	Constant bit rate
CTMC	Continuous-time Markov
DSCR	Debt service coverage ratio
DARP	Dial-a-ride problems
DSGA	Driver social group architecture
DRL	Deep reinforcement learning
DTS-MDP	Double time-scale Markov decision process
DDPG	Deep deterministic policy gradient
DQN	Deep Q networks
DDP4V	Data dissemination protocol for vehicular networks
ECPP	Efficient conditional privacy preservation
FTP	File transfer protocol
GSB	Group signature based
HOPs	Hop counts
ITS	Intelligent transportation system
IoT	Internet of things
I2I	Infrastructure to infrastructure
JSS	Joint socialness score
KPSD	Key-insulated pseudonym self delegation
KD	Knowledge-driven
LSTM	Long-short term memory
MANET	Mobile ad hoc network
MFD	Macroscopic fundamental diagram
MCN	Maximum connectivity number
MaxMin	Maximize the minimum distance
MaxSum	Maximize the total distance
MaxMinSum	Maximize the minimum aggregate dispersion
MinDiff	Minimize the gap between max and mini of aggregate dispersion
MWT	Mean waiting time
NS-2	Network simulator version 2
NFV	Next forwarder vehicle
OBUs	On-board units
PSR	Packet sending rate
PSO	Particle swarm optimization
QoS	Quality of service
RSUs	Roadside units
RFID	Radio frequency identification
SGA	Social group architecture
SUMO	Simulation of urban mobility
SAMA	Secure and anonymous mutual authentication
SPVs	Service providing vehicles
SDN	Software-defined networking
TCP	Transmission control protocol
UDP	User datagram protocol
VANETs	Vehicular ad hoc networks
VEC	Vehicular edge computing
V2V	Vehicle to vehicle
V2I	Vehicle to infrastructure
V2X	Vehicle to everything
VLC	Visible light communication
VSGA	Vehicle social group architecture
VN	Virtual nodes
VNLayer	Virtual node layer
VNAODV	Virtual nodes ad-hoc on-demand distance vector
VBR	Variable bit rate
WiMAX	Worldwide interoperability for microwave access
WSS	Within-cluster sum of squares
UAV	Unmanned aerial vehicle
